# Early adolescent aggression predicts antisocial personality disorder in young adults: a population-based study

**DOI:** 10.1007/s00787-018-1198-9

**Published:** 2018-07-17

**Authors:** Alyce M. Whipp, Tellervo Korhonen, Anu Raevuori, Kauko Heikkilä, Lea Pulkkinen, Richard J. Rose, Jaakko Kaprio, Eero Vuoksimaa

**Affiliations:** 1 Institute for Molecular Medicine Finland, University of Helsinki, PL 20 (Tukholmankatu 8 B), 00014 Helsinki, Finland; 2 Department of Public Health, University of Helsinki, Helsinki, Finland; 3 Department of Adolescent Psychiatry, Helsinki University Central Hospital, Helsinki, Finland; 4 Department of Psychology, University of Jyvaskyla, Jyvaskyla, Finland; 5 Department of Psychological and Brain Sciences, Indiana University, Bloomington, IN USA

**Keywords:** Adolescent, Aggression, Antisocial personality disorder, Population-based, Psychiatric prediction

## Abstract

Modestly prevalent in the general population (~ 4%), but highly prevalent in prison populations (> 40%), the diagnosis of antisocial personality disorder (ASPD) involves aggression as one of several possible criteria. Using multiple informants, we aimed to determine if general aggression, as well as direct and indirect subtypes, assessed in early adolescence (ages 12, 14) predict young adulthood ASPD in a population-based sample. Using data from a Finnish population-based longitudinal twin cohort study with psychiatric interviews available at age 22 (*N* = 1347), we obtained DSM-IV-based ASPD diagnoses. Aggression measures from ages 12 (parental and teacher ratings) and 14 (teacher, self, and co-twin ratings) were used to calculate odds ratios (OR) of ASPD from logistic regression models and the area under the curve (AUC) from receiver operating characteristic curve analysis. Analyses were adjusted for sex, age, and family structure. All informants’ aggression ratings were significant (*p* < 0.05) predictors of ASPD (OR range 1.3–1.8; AUC range 0.65–0.72). Correlations between informants ranged from 0.13 to 0.33. Models including two or more aggression ratings, particularly age 14 teacher and self ratings, more accurately predicted ASPD (AUC: 0.80; 95% confidence interval 0.73–0.87). Direct aggression rated by all informants significantly predicted ASPD (OR range 1.4–1.9), whereas only self-rated indirect aggression was significantly associated with ASPD (OR = 1.4). Across different informants, general and direct aggression at ages 12 and 14 predicted ASPD in a population-based sample. Psychiatric, social, and parenting interventions for ASPD prevention should focus on children and adolescents with high aggression levels, with an aim to gather information from multiple informants.

**Electronic supplementary material:**

The online version of this article (10.1007/s00787-018-1198-9) contains supplementary material, which is available to authorized users.

## Introduction

Antisocial personality disorder (ASPD) is prevalent in approximately 4% of the general population [1, 2], but is roughly ten times more common among the prison population [3]. Aside from the high association with crime, this serious disorder may have other long-reaching consequences for individuals, their families, and society, such as loss of employment, housing, or relationships, and substance abuse problems [1, 4, 5]. Although, based on Diagnostic and Statistical Manual of Mental Disorders (DSM) [6] criteria, an individual must be at least 18 years old to be diagnosed, the roots of ASPD are evident in childhood and adolescence.

Individuals with ASPD have to have shown, since at least the age of 15, a pervasive pattern of violating the rights of others, often without feelings of remorse, including displays of antisocial behavior and conduct problems [4, 7]. Criteria for ASPD diagnosis in both the DSM-IV and DSM-5 include deceitfulness, callousness, hostility, irresponsibility, impulsivity, and risk taking [6, 8], with symptoms from 3 or more categories fulfilling the diagnostic criteria. As a heterogenous, multidimensional disorder, predictive research regarding ASPD is complicated.

While investigating antisocial behavior and conduct problems in the context of ASPD has yielded important findings, these behavior problems are already quite serious. Although aggression has been shown to predict antisocial behavior and conduct problems in the general population [7, 9, 10], the independent connection between aggression and ASPD has been less well studied. Consequently, little is known of the predictive features of different subtypes of aggression to ASPD [11]. Widespread subtyping of aggressive behavior is formulated with respect to the underlying goal and includes reactive (e.g., impulsively, defensively responding to provocation) and proactive aggression (e.g., used as an instrumental means to secure goods from others or to dominate them), or direct and indirect aggression. Reactive, proactive and direct aggressive behaviors are often better understood in the context of antisocial behavior and ASPD than indirect aggression (e.g., spreading rumors and socially ostracizing someone) for example [11, 12]. Furthermore, within antisocial behavior and conduct problems there is often a distinction made between aggressive and non-aggressive (i.e., rule-breaking) behaviors [6, 8, 13, 14]. Although aggression is one potential component of ASPD, we lack knowledge regarding whether the association is strong enough, and whether indirect aggression can be utilized, for ASPD prediction. With prediction comes the possibility for intervention; indeed, intervening on aggression in childhood can possibly reduce future negative outcomes [15, 16].

Studies of ASPD prediction in the general population have been rare [17, 18]. Typically, clinical or prison-based samples are employed to investigate ASPD [19, 20], and because ASPD occurs more frequently in males [1, 2], these samples often include only males [21–23]. Additionally, because ASPD diagnostics are difficult to perform on epidemiological samples, the outcome utilized by many prediction studies is antisocial behavior [7, 9, 10], a measure that is non-diagnostic and more broad than ASPD, but more feasible to capture.

Using a population-based sample, the aims of our study were to determine: 1) if the level of aggression assessed in early adolescence predicts ASPD in young adulthood, 2) if both direct and indirect aggression subtypes can predict ASPD, and 3) to what extent different sources of aggression ratings predict ASPD.

## Methods

### Participants

Participants were from the FinnTwin12 study, a population-based cohort tracking the behavioral development and health habits among all Finnish twins born between 1983 and 1987 (Online Resource 1) [24–26]. Families with twins were identified through the Finnish Central Population Registry with twin pairs enrolled between the ages of 11 and 12. The initial enrollment response rate was 87% (*N* = 5600 twins) and remained between 85 and 90% for all data collection waves. Questionnaires were collected from individual twins at tightly controlled age-bands in pre- and early adolescence [ages 11–12, 14 and 17; means (standard deviations (SD)]: 11.42 (0.30), 14.05 (0.08), 17.63 (0.26), respectively). Additionally, parents and a teacher of each twin at age 12 completed questionnaires regarding the twins, as well as a teacher of each twin at age 14.

Furthermore, a subset of the twins, from which the current study sample comes, was more intensively studied. This subsample of 1035 families was mostly comprised of randomly selected twins across all study years, while about a third were selected based on parental self-reports of elevated risk for alcohol problems. Previous investigation has shown that this modest enrichment of at-risk families did not systematically affect substance use or behavioral problems among the twins through adolescence [27]. Additional assessments of this more intensively studied subsample included one-on-one psychiatric interviews at ages 14 (mean = 14.2, SD = 0.15; *n* = 1852, response rate 89.5%) and 22 (mean = 22.4, SD = 0.70; *n* = 1347, response rate 73.0%). At the time of the age 14 interview, individuals completed an additional questionnaire which included self and co-twin assessments of emotional and behavioral problems.

### Ethical considerations

All waves of the study’s data collection were approved by the ethical committee of the Helsinki and Uusimaa University Hospital District and Indiana University’s Institutional Review Board. Parents provided informed consent for the twins at ages 12 and 14, and the twins themselves provided written informed consent at age 22.

### Measures

#### Antisocial personality disorder

At age 22, the psychiatric interviews from the intensively studied subsample were conducted using a translated version of the Semi-Structured Assessment for the Genetics of Alcoholism (SSAGA). This validated multi-diagnostic instrument gathers information on a number of psychiatric disorders, including ASPD, based on DSM-IV criteria [28, 29]. In this study, 15 behaviors (e.g., purposeful harm of animals, forgery, arson, neglecting family duties) corresponding to the 7 criteria of DSM-IV ASPD were available. If an individual had a history of conduct problems before age 15 (assessed through the SSAGA instrument at the age 22 interview) and endorsed 3 or more ASPD behaviors, he/she was considered to have ASPD. Of note, all psychiatric interviews took place among presently non-incarcerated individuals.

#### Aggression and other ratings

We utilized the 37-item modified Multidimensional Peer Nomination Inventory (MPNI), which is comprised of 3 factors, giving rise to 8 scales: behavioral problems [aggression (6 items), impulsivity-hyperactivity (7 items), inattention (4 items)], emotional problems [social anxiety (2 items), depression (5 items)], and adjustment (12 items) [30, 31]. The six aggression questions consist of both direct (four questions) and indirect (two questions) aggression dimensions. For each question (e.g., “Does the child tease smaller/weaker children?”), the informant rated the child in question on a scale from 0 (does not fit the child at all) to 3 (fits the child very well). If an ambivalent response was given (e.g., an informant rated both 1 and 2), the mean of the two values was utilized. Aggression summary scores were created by averaging the available question responses: total aggression mean score (all six aggression questions), direct aggression mean score (only the four direct aggression questions), and indirect aggression mean score (only the two indirect aggression questions). One missing response was allowed for the total and direct scores, however, the indirect score required both responses to calculate a value. For this study, we used MPNI aggression ratings before the age of 15 (the age when children in Finland are legally considered responsible for their actions, as well as the ASPD cut-off age for conduct problems having to have begun). Aggression was rated by the parents (60% rated by mothers alone, 37% by both parents together, 3% other or rater data missing) at the age of 12, by a teacher at ages 12 and 14, and by the child him/herself and their co-twin at age 14.

### Statistical analysis

All statistical analyses were performed using Stata 13.0 (StataCorp, College Station, TX, USA) and all were adjusted for the clustered nature of the twin data. Statistical significance for all analyses was considered to be *p* < 0.05. Total aggression mean scores of the participants from the different informants were compared for those with and without ASPD, both with the sexes combined and separated. Additionally, direct and indirect aggression score mean differences between the sexes were compared. Mean differences were tested for significance using the adjusted Wald test. Effect sizes were calculated as Cohen’s *d*. Pearson correlations were calculated between the total aggression scores of different informants, as well as between direct and indirect aggression scores from each informant. Additionally, to obtain an initial correlation of ASPD and aggression, a continuous ASPD behavior count was used for correlations with total aggression scores from different informants.

Before running logistic regression models of aggression with ASPD, we first established a base model with only sex and exact age at the time of ASPD assessment as predictors of ASPD. Following this analysis, initial aggression models were performed with only one main predictor—one informant’s total aggression scores—in the model at a time, adjusted for sex and age. In these logistic regression models, the aggression scores (total, direct, indirect) from each informant were standardized (mean = 0, SD = 1) to allow for direct comparison. Additionally, receiver operating characteristic (ROC) curves were generated for the models and the area under the ROC curve (AUC) was calculated with 95% confidence intervals (CI) to demonstrate the predictive utility of the different informants’ aggression ratings in ASPD prediction [32]. We also tested for possible sex interactions with the total aggression scores from the different informants.

Following the initial aggression models, models including multiple aggression ratings were considered. First, for each informant, low to moderately correlated (*r* < 0.65) direct and indirect aggression scores were entered into logistic regression models simultaneously to examine whether separating the total aggression rating into subtypes clarified which type of aggression was contributing most to the ASPD prediction. Second, low or moderately correlated total aggression scores from different informants were entered into logistic regression models simultaneously to examine whether additional assessments of aggression improved ASPD prediction.

Finally, a series of supplemental analyses were performed to confirm the robustness of our findings. In brief, first, MPNI impulsivity-hyperactivity (hereafter, impulsivity) ratings analyses were run in a similar manner to the main aggression ratings analyses (mean differences of impulsivity between ASPD and non-ASPD cases, correlations between informants, univariate models with only impulsivity ratings), as well as residual models where the effect of impulsivity was removed from aggression and vice versa, and a test to see if adding impulsivity ratings into the multi-rater aggression model with the highest AUC value improved ASPD prediction. Second, an additional alternative model was explored where MPNI social anxiety ratings (instead of aggression) were used as the main predictor. Third, we modified the ASPD definition: endorsing at least one, two, or four behaviors, or removing sub-clinical ASPD cases (one or two behaviors) from analyses. Fourth, we created residual scores for direct and indirect aggression that removed the effect of one on the other. Finally, we examined attrition by comparing mean differences in age 12 parent and teacher ratings among all available FinnTwin12 participants and in those who participated in age 22 psychiatric interviews (along with several additional subgroup combinations) to confirm that our study sample of individuals were neither more nor less aggressive than other FinnTwin12 participants.

## Results

### Main analyses

The final sample included 1347 individuals (53% female), of which 5% (*n* = 67) met diagnostic criteria for ASPD (64% male). Total aggression scores for those with and without ASPD were significantly different across all informants (Table 1). Furthermore, most mean differences remained significant when the sexes were analyzed separately. Regarding the informant differences in direct aggression scores, males consistently had significantly higher direct aggression scores than females (Online Resource 2). For indirect aggression, however, trends were less uniform.

**Table 1:** Total aggression mean score comparisons from different informants by ASPD diagnosis and sex

Informant (age of participant)	Sex	ASPD *N*/total *N*	No ASPD	ASPD	*p* value^a^	Effect Size^b^
Parent (12)	Combined	64/1278	0.58	0.71	0.008	0.32
	Female	21/668	0.54	0.65	0.094	
	Male	43/610	0.62	0.74	0.074	
Teacher (12)	Combined	65/1302	0.61	1.00	<0.001	0.61
	Female	23/690	0.52	0.69	0.181	
	Male	42/612	0.72	1.17	<0.001	
Teacher (14)	Combined	49/1013	0.29	0.72	<0.001	0.94
	Female	17/546	0.22	0.50	0.053	
	Male	32/467	0.38	0.83	<0.001	
Self (14)	Combined	63/1312	0.46	0.75	<0.001	0.80
	Female	24/695	0.39	0.63	0.007	
	Male	39/617	0.54	0.82	<0.001	
Co-Twin (14)	Combined	54/1208	0.57	0.91	<0.001	0.69
	Female	20/647	0.48	0.76	0.033	
	Male	34/561	0.67	0.99	0.002	

^a^Mean differences evaluated using adjusted Wald test

^b^Cohen’s *d*

Between the different informants, correlations of the total aggression ratings ranged from low to moderate (*r* range 0.13–0.33), with the highest correlations being between the teacher ratings at age 12 and 14 (*r* = 0.31) and the age 14 self and co-twin ratings (*r* = 0.33) (Table 2). Additionally, correlations between the direct and indirect aggression subscales of each informant were moderate (teacher at age 12 *r* = 0.62, all others ranged 0.34–0.52).

**Table 2:** Pearson correlations between different informants’ total aggression scores

Informant (age of participant)	Parent (12)	Teacher (12)	Teacher (14)	Self (14)	Co-Twin (14)
Parent (12)	–				
Teacher (12)	0.26	–			
Teacher (14)	0.15	0.36	–		
Self (14)	0.13	0.18	0.18	–	
Co-Twin (14)	0.27	0.22	0.21	0.33	–

All correlations, *p* < 0.001

*N* range for correlations, 918–1270

**Fig. 1:**
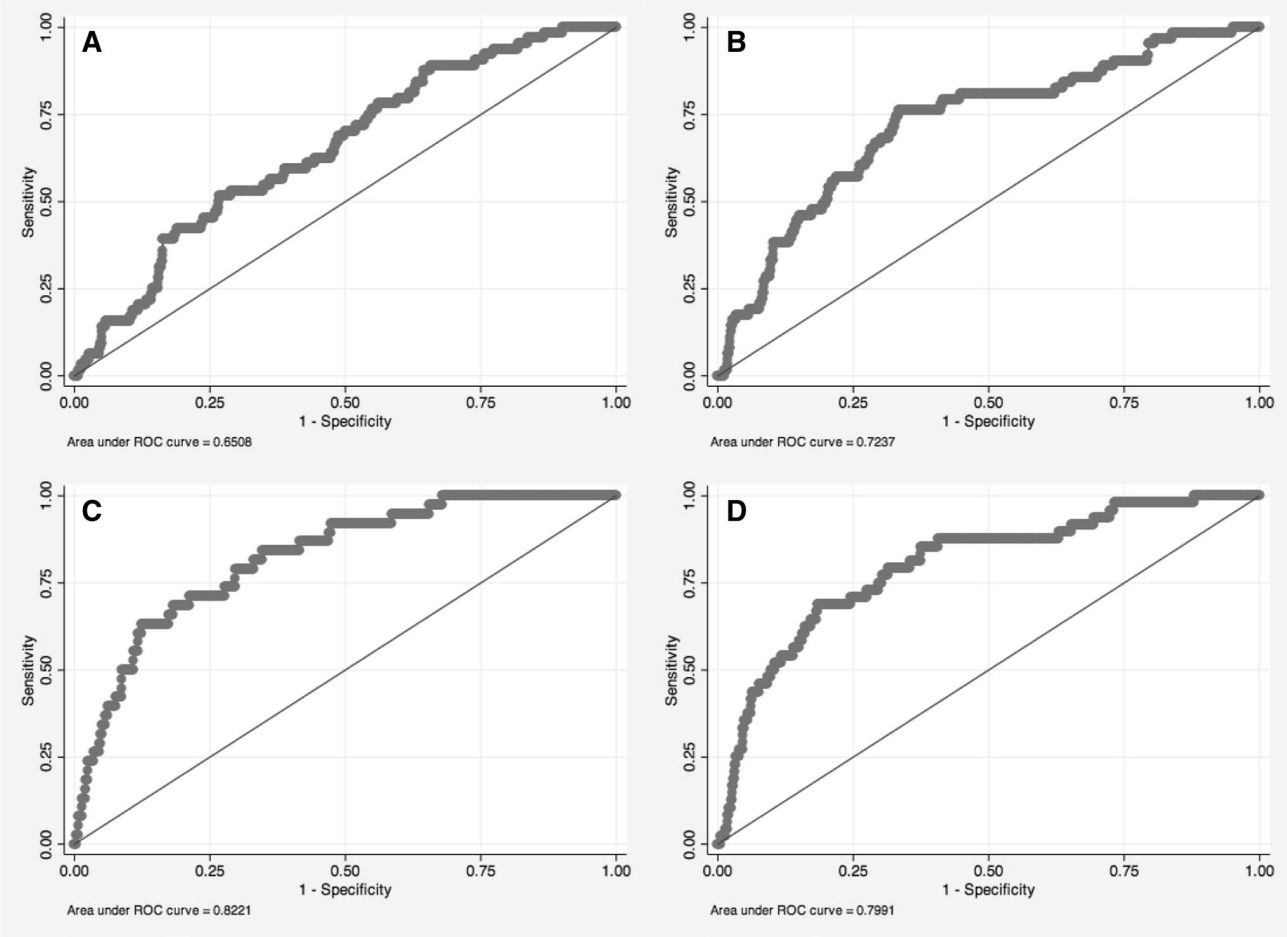
a-d Receiver operating characteristic (ROC) curves of select total aggression score logistic models. **a** Parent rating (12) only model. **b** Self rating (14) only model. **c** All 5 ratings (parent-12, teacher-12, teacher-14, self-14, co-twin-14) model. **d** Teacher (14) and self (14) model

Logistic regression of the base model (sex and age as only predictors) showed sex significantly predicted ASPD [odds ratio (OR) 2.1; 95% CI 1.2, 3.4], with AUC value of 0.60 (95% CI 0.54, 0.66). In initial aggression models, total aggression scores from the different informants all significantly predicted ASPD (Table 3), with ORs ranging from 1.3 to 1.8 and 95% CIs of all models overlapping for all aggression rating ORs. AUC values ranged from 0.65 to 0.72, with all 95% CIs also overlapping (Table 3; Fig. 1a, b). Finally, while sex was also a significant predictor of ASPD in the parental and teacher age 12 aggression ratings models (OR 2.2 and 1.8, respectively), no sex-by-aggression interactions on ASPD were observed in any of the total aggression score models (*p* value range 0.27–1.0).

**Table 3:** Logistic regression models and AUC values for ASPD prediction by each informant’s total aggression score or separate direct and indirect aggression scores, adjusted for sex and age

Informant (age of participant)	Total aggression models						Direct + indirect aggression models					
	*N*	Model variable	OR	95% CIs	AUC	95% CIs	*N*	Model variable	OR	95% CIs	AUC	95% CIs
Parent (12)	1278				0.65	0.59, 0.72	1277				0.66	0.60, 0.72
		Total AGG	1.3*	1.1, 1.6				Direct AGG	1.4*	1.1, 1.8		
		Sex	2.2*	1.3, 3.8				Indirect AGG	0.9	0.7, 1.1		
								Sex	2.0*	1.2, 3.5		
Teacher (12)	1302				0.69	0.62, 0.75	1292				0.70	0.63, 0.76
		Total AGG	1.6*	1.3, 1.9				Direct AGG	1.9*	1.4, 2.6		
		Sex	1.8*	1.1, 3.0				Indirect AGG	0.8	0.6, 1.2		
								Sex	1.4	0.8, 2.5		
Teacher (14)	1013				0.72	0.65, 0.80	994				0.73	0.65, 0.81
		Total AGG	1.7*	1.4, 2.1				Direct AGG	1.7*	1.2, 2.3		
		Sex	1.7	0.9, 3.2				Indirect AGG	1.1	0.7, 1.5		
								Sex	1.6	0.8, 3.2		
Self (14)	1312				0.72	0.66, 0.79	1307				0.72	0.66, 0.79
		Total AGG	1.8*	1.5, 2.2				Direct AGG	1.5*	1.2, 1.8		
		Sex	1.4	0.8, 2.4				Indirect AGG	1.4*	1.1, 1.7		
								Sex	1.4	0.8, 2.4		
Co-Twin (14)	1208				0.69	0.62, 0.76	1201				0.69	0.62, 0.76
		Total AGG	1.6*	1.3, 2.0				Direct AGG	1.7*	1.3, 2.2		
		Sex	1.6	0.9, 2.9				Indirect AGG	1.0	0.8, 1.3		
								Sex	1.5	0.8, 2.6		

*AGG* aggression, *ASPD* antisocial personality disorder, *AUC* area under the (receiver operating characteristic) curve, *CI* confidence interval, *OR* odds ratio

*p < 0.05

Analyses with multiple aggression ratings provided additional ASPD prediction clarity. First, because correlations between an informant’s direct and indirect aggression ratings were moderate, the two aggression subscores were entered into models simultaneously for each informant, adjusted for sex and age. Direct aggression was significant in all models (OR range 1.4–1.9), while indirect aggression was only significant in the self-rated aggression model (OR = 1.4) (Table 3). AUC values for all models ranged 0.66–0.73.

Furthermore, since all correlations between the aggression ratings from the different informants were low to moderate, logistic regression models were performed using combinations of total aggression scores from different informants, adjusted by sex and age, to predict ASPD (see Table 4 for combinations tested). In general, AUC values for these models were higher than models with only one informant’s aggression rating included; the highest AUC values were seen in the model with all age 14 ratings and the model with all 5 ratings (AUC: 0.81 and 0.82, respectively) (Table 4, Fig. 1c). In both of these models, only the ORs for the age 14 teacher and self ratings were significant, therefore, an additional model was run with only these two ratings included. In this model, the ORs for the teacher and self ratings were significant and robust (OR 1.6 and 1.9, respectively), and the AUC value remained high (AUC = 0.80) (Fig. 1d). Additionally, to confirm that the varying sample sizes of the different informant combination models were not driving the differences seen in AUC values, all combinations tested were rerun restricted to the 847 participants in the model with the best AUC value (model with all 5 ratings): all results remained similar (data not shown).

**Table 4:** Multiple informant logistic regression model combinations of total aggression scores for ASPD prediction, adjusted by sex and age

Model	*N*	Model variable	OR	95% CIs	AUC	95% CIs
PR12 + TR12	1240	PR12	1.2	0.9, 1.4	0.69	0.62, 0.75
		TR12	1.5*	1.2, 1.8		
		Sex	1.9*	1.1, 3.3		
TR14 + SR14 + TWR14	907	TR14	1.6*	1.3, 2.1	0.81	0.74, 0.88
		SR14	1.8*	1.4, 2.4		
		TWR14	1.1	0.8, 1.6		
		Sex	1.2	0.5, 2.5		
TR12 + TR14	982	TR12	1.3*	1.0, 1.7	0.74	0.67, 0.81
		TR14	1.6*	1.3, 2.0		
		Sex	1.7	0.9, 3.2		
PR12 + TR12 + TR14	939	PR12	1.2	0.9, 1.6	0.74	0.66, 0.82
		TR12	1.2	0.9, 1.6		
		TR14	1.6*	1.3, 2.0		
		Sex	1.9	1.0, 3.6		
SR14 + TWR14	1191	SR14	1.7*	1.3, 2.1	0.74	0.67, 0.81
		TWR14	1.4*	1.1, 1.8		
		Sex	1.3	0.7, 2.3		
PR12 + TR12 + TR14 + SR14 + TWR14	847	PR12	1.2	0.9, 1.6	0.82	0.76, 0.88
		TR12	1.3	1.0, 1.8		
		TR14	1.5*	1.2, 1.9		
		SR14	1.7*	1.3, 2.2		
		TWR14	1.1	0.8, 1.5		
		Sex	1.3	0.6, 2.7		
TR14 + SR14	992	TR14	1.6*	1.3, 2.0	0.80	0.73, 0.87
		SR14	1.9*	1.5, 2.4		
		Sex	1.2	0.6, 2.4		

*PR* parent rating, *TR* teacher rating, *SR* self rating, *TWR* co-twin rating, *ASPD* antisocial personality disorder, *CI* confidence interval, *OR* odds ratio, *AUC* area under the (receiver operating characteristic) curve

*p < 0.05

### Supplemental analyses

In the additional impulsivity ratings analyses, significant mean differences in impulsivity between ASPD and non-ASPD cases were seen (for males, as well as females for age 14 ratings) (Online Resource 3). Most correlations between informants and aggression and impulsivity scores were low to moderate (*r* range 0.10–0.54), with teacher age 12 and 14 aggression and impulsivity scores having higher correlations (0.73 and 0.68, respectively). In univariate impulsivity models, all informant impulsivity scores significantly predicted ASPD. However, in impulsivity residual models (i.e., each impulsivity measure uncorrelated with corresponding aggression measure), only self and co-twin age 14 ratings still predicted ASPD. Furthermore, in aggression residual models (i.e., each aggression measure uncorrelated with corresponding impulsivity measure), all of the age 14 aggression ratings (teacher, self, co-twin) still predicted ASPD. Finally, when comparing a multi-variate, multi-rater aggression and impulsivity model to the multi-rater aggression model with all 5 ratings simultaneously in the model, the teacher 14 and self 14 aggression scores were still the only significant predictors of ASPD and AUC values were the same (0.82), though a likelihood ratio test (comparing these two ‘best’ models) indicated the aggression + impulsivity model was significantly better (*p* = 0.015).

In the alternative social anxiety ratings models, none of the social anxiety ratings were significant predictors of ASPD and all ORs were below 1.0 (Online Resource 4). The supplemental models using modified definitions of ASPD all produced similar ORs and all were significant (Online Resource 5). AUC values were also similar. Supplemental analyses using either the direct or indirect residual scores produced results similar to when both aggression subscores were modeled simultaneously. In the direct aggression residual score models, the ORs were all significant, with AUC values slightly lower than main models (Online Resource 6). In the indirect aggression residual score models, only the self rating OR was significant. Attrition analyses showed the mean differences in aggression values were not significantly different between those in our final sample and those not in our final sample (Online Resource 7).

## Discussion

This study indicated that measurements of general aggression, gathered in early adolescence, are able to predict a serious adult personality disorder, ASPD, in young adulthood in both sexes within a population-based sample. Most odds ratios in single-informant aggression models were greater than 1.5, suggesting that for those informants’ ratings of aggression (at the participant’s age at collection), each standard deviation unit increase in observed aggressive behavior corresponded to at least a 50% increased odds of having an ASPD diagnosis in young adulthood. AUC values also indicate modest levels of predictive utility. Furthermore, the results were similar regarding the direct aggression subtype, whereas there was less evidence for an independent effect of indirect aggression on ASPD. Aggression ratings from all informants significantly predicted later ASPD. However, when multiple informants were included in the model, the prediction accuracy improved.

Prediction of ASPD in a population-based sample using a measure of general aggression in pre- and early adolescence is an important addition to the literature. Our study provided 8–10 years of longitudinal follow-up from the time of the aggression ratings to the time of ASPD assessment. Furthermore, age ranges for the data collection at ages 12 and 14 were narrow, providing aggression measures that were as developmentally specific as possible. To our knowledge, only two other studies have investigated ASPD prediction using a population-based sample. Caspi, Moffitt, Newman, and Silva [17] utilized examiner observations of behavior among a birth cohort of 3-year-old children and found that under-controlled (impulsive, restless, or distractable) children were more likely to meet diagnostic criteria for ASPD at age 21. Copeland, Shanahan, Costello, and Angold [18] demonstrated the ability of childhood and adolescent psychiatric disorders (e.g., conduct disorder) to predict adult psychiatric disorders (e.g., ASPD). These studies each had their specific research questions regarding ASPD, as does ours. In aiming to examine how much the behavior of aggression was capable of predicting ASPD, we did not adjust for all possible ASPD predictors. We were, however, able to show that aggression does significantly predict ASPD, with significance remaining for teacher, self, and co-twin (age 14) ratings after the effect of impulsivity on aggression was removed. We further show that impulsivity provides additional significant ASPD prediction for self and co-twin ratings after the effect of aggression on impulsivity was removed, and that social anxiety does not appear to predict ASPD.

Our results also offer evidence of ASPD prediction from the aggression subtypes, direct aggression in particular. In all models, direct aggression ratings significantly predicted ASPD at levels similar to those of the total aggression models, with similar AUC values as well. This may indicate direct aggression as a specific subtype closely associated with ASPD, however, these results may also reflect the higher proportion of direct aggression items comprising the aggression scale. Regarding indirect aggression, while our power is perhaps low due to the indirect aggression score coming from only two questions, ratings from at least one informant (self, age 14), significantly predicted ASPD. These aggression subtype findings were consistent whether the two aggression subtypes were modeled simultaneously or as residual scores separately. Of note, while the odds ratios were similar between the two models, the AUC values were consistently higher when the subtypes were modeled simultaneously. Although the two subtypes are correlated, perhaps unique differences between the subtypes convey additional value in improving the strength of the prediction when simultaneously modeled.

Sex differences existed, to some extent, in our study. Our ASPD cases included about two-thirds males, which is typical [1]. The base model with only sex and age showed that sex is a significant predictor of ASPD, however, the AUC value was quite low, indicating that sex accounts only for a small portion of the variance in ASPD. Significant sex differences were also observed in the initial aggression models with age 12 aggression ratings, as well as multiple informant models that included parental ratings, indicating an increased odds for males to have ASPD in young adulthood. However, findings revealed there were no sex-by-aggression interactions, meaning that aggression ratings did not differentially affect the prediction of ASPD between the sexes. Larger samples may be needed, however, for detecting sex-specific patterns of these associations.

All informants’ total aggression ratings were able to successfully predict ASPD in single-informant models. While trends suggested parental aggression ratings to be the least robust and teacher and self ratings at age 14 the most predictive, in fact, the confidence intervals of all odds ratios and AUCs overlapped. Furthermore, it is important to remember that informant data were collected at either age 12 or 14, thus, the age 14 informant ratings may appear to be more predictive simply because they are temporally closer to the ASPD diagnosis. Differences in ratings could also partly be due to situation-specific behavior differences in the child [33, 34] or to different informants being able to better observe or identify certain behaviors. Compared to other behaviors, however, aggression is typically better agreed upon by informants [35–37].

Although all informants were separately able to significantly predict ASPD, our results do support the inclusion of multiple informants in ASPD prediction models to increase the predictive utility. The relatively low correlations of aggression ratings between informants, a typical finding in the literature [38], indicated the ability to add more than one informant rating into the same model. With multiple informants in one model, AUC values rose. Thus, the more information available from multiple informants, the more accurate the ASPD prediction. In line with other studies encouraging multiple raters [34, 36, 37], our results offer yet more motivation to include more than one informant when collecting behavioral ratings.

This study has many strengths and some notable limitations. Strengths include the high participation rate of the large population-based sample, the 8–10 years of follow-up time between aggression ratings in early adolescence and psychiatric data collection in young adulthood, and the fact that attrition was not selective on aggression ratings. Additionally, while the dataset is comprised of twins, previous studies have shown the twins to be representative of the population in general, being no different than their singleton peers regarding externalizing and internalizing problems [39]. Important to note, however, is that while our data came from a population-based sample, the current study sample was derived from a more intensively studied subset of that population. Furthermore, while this is a sample based on a general population, it is necessary to understand the culture and context of the population utilized. Finland has a low incarceration rate compared to most of the world, and has had specific policies in place for decades to reduce the adolescent prison population. Thus, our sample may be comprised of a slightly different mix of individuals than countries with high incarceration rates. Since we do not have legal data on the participants, we are unable to examine this aspect further in our sample.

Additionally, our power for subgroup-specific patterns in ASPD prediction (e.g., male vs. female) may have been reduced due to having 67 ASPD cases (among 1347 participants). This 5% prevalence, however, is among typical population prevalence rates [1, 2], and relatively low case numbers is the natural consequence of utilizing a population-based sample for studies of uncommon conditions. Another limitation concerning power is that Marcus, Lilienfeld, Edens, and Poythress [40] suggest that ASPD exists on a continuum and that dichotomization—ASPD versus non-ASPD—could lead to a reduction in power. However, our supplemental alternative ASPD cut-off analysis showed that adjusting the ASPD diagnostic cut-off did not significantly affect the strength of the odds ratios, though AUC values fluctuated somewhat between models. Additionally, removal of sub-clinical ASPD cases also did not significantly improve odds ratios or AUC values. Thus, the main model indications that increasing levels of aggression predict ASPD hold no matter how we dichotomize ASPD. Finally, we also acknowledge that supplemental analyses and results related to social anxiety may reflect having only two items, rather than definitive lack of social anxiety association with ASPD.

A final consideration is that the findings of this initial investigation could be expanded to utilize the genetic information inherent in twin cohorts to better understand the genetic underpinnings of the relationship between aggression and ASPD. This would fit well within the aims of the ACTION (Aggression in Children: unraveling gene-environment interplay to inform Treatment and InterventiON strategies; http://www.action-euproject.eu/) consortium, of which the FinnTwin12 study is a part.

Our results indicate that aggression levels in a general pre- and early adolescent population can significantly predict, with useful predictive utility, a serious psychiatric outcome (ASPD) in young adulthood in both sexes. Study findings also lend further support for the collection of behavioral ratings from multiple informants to improve the predictive utility of externalizing disorders. Furthermore, the direct aggression subtype can also consistently and significantly predict ASPD. Taken together, these findings suggest that focusing interventions on children and adolescents with higher aggression levels could reduce future ASPD cases.

## Electronic supplementary material

Below is the link to the electronic supplementary material.

Supplementary material 1 (PDF 645 kb)
