# Supplementary material for: Early adolescent aggression predicts antisocial personality disorder in young adults: a population-based study
Source: Eur Child Adolesc Psychiatry. 2018 Jul 17;28(3):341–50. doi: 10.1007/s00787-018-1198-9 (PMC6407741; doi:10.1007/s00787-018-1198-9)
Supplement: Supplementary file 1 — Supplementary material 1 (PDF 645 kb) [file 787_2018_1198_MOESM1_ESM.pdf]

Online Resource 1. Flow chart of FinnTwin12 study waves and data collection included in the current study

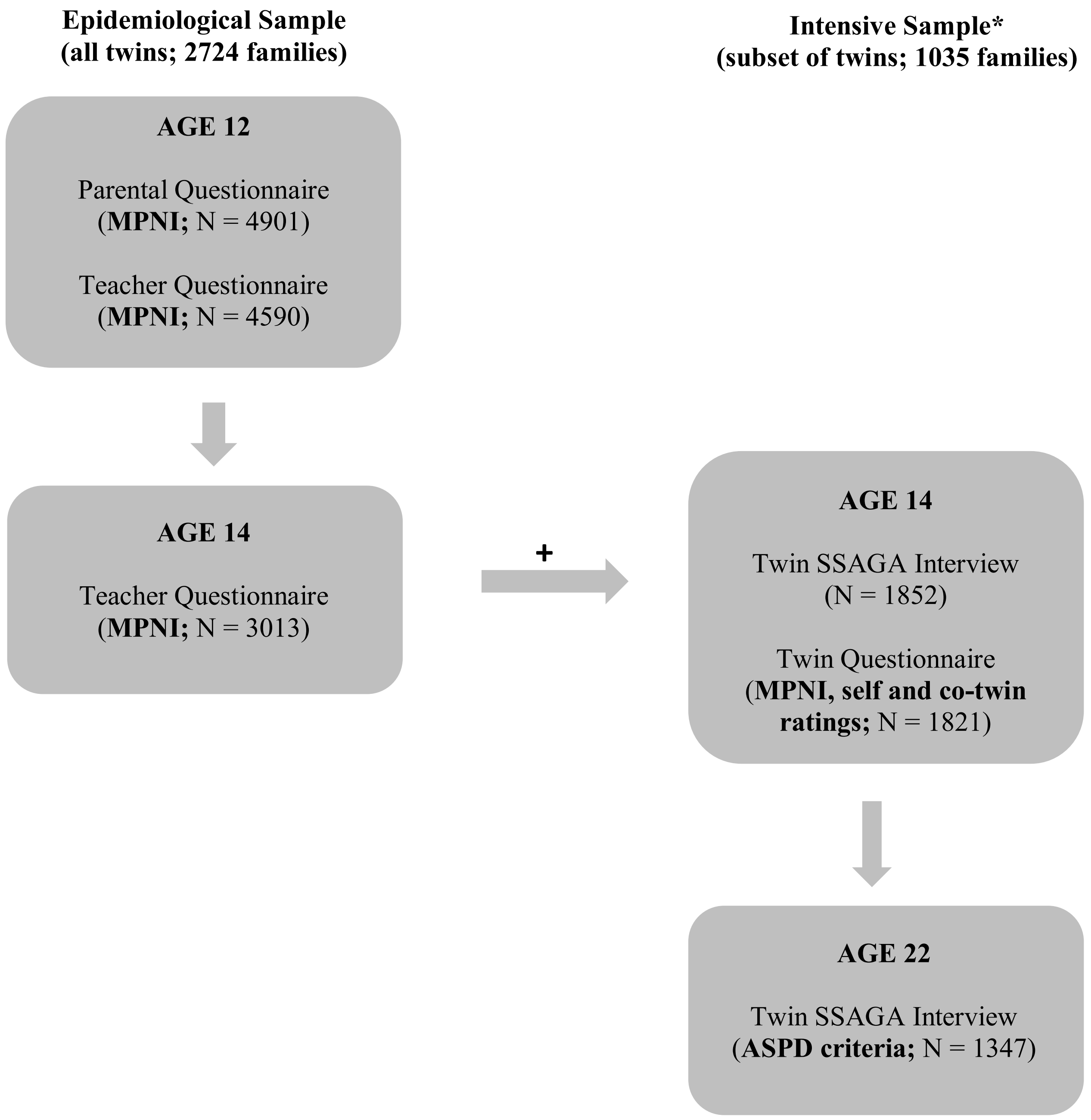

*Data for intensive sample participants includes both epidemiological sample data collection materials as well as intensive sample data collection materials

Abbreviations: ASPD=Antisocial Personality Disorder; MPNI=Multidimensional Peer Nomination Inventory; SSAGA=Semi-Structured Assessment for the Genetics of Alcoholism

Online Resource 2. Direct and indirect mean aggression score comparisons from different informants by sex

Informant (age of participant)	Sex	N	Direct Aggression Mean (SD)	N	Indirect Aggression Mean (SD)
Parent (12)					
	Female	668	0.60 (0.43)*	668	0.45 (0.50)*
	Male	610	0.76 (0.49)*	609	0.36 (0.48)*
Teacher (12)					
	Female	690	0.47 (0.59)*	686	0.64 (0.70)*
	Male	615	0.86 (0.77)*	606	0.55 (0.66)*
Teacher (14)					
	Female	549	0.20 (0.37)*	541	0.27 (0.47)
	Male	467	0.47 (0.61)*	453	0.28 (0.49)
Self (14)					
	Female	695	0.43 (0.35)*	694	0.33 (0.45)*
	Male	618	0.63 (0.43)*	613	0.41 (0.50)*
Co-Twin (14)					
	Female	648	0.49 (0.46)*	642	0.47 (0.61)
	Male	561	0.77 (0.56)*	559	0.53 (0.62)

*Mean difference between males and females is significant at $p<0.05$
Abbreviation: SD=standard deviation

Online Resource 3. Impulsivity analyses

Table: Impulsivity mean score comparisons from different informants by ASPD diagnosis and sex

Informant (age of participant)	Sex	No ASPD	ASPD	P-value[a]	Effect Size[b]
Parent (12)					
	Combined	0.71	0.94	0.004	0.23
	Female	0.64	0.78	0.206	
	Male	0.78	1.01	0.027	
Teacher (12)	Combined	0.66	1.14	<0.001	0.48
	Female	0.45	0.51	0.711	
	Male	0.89	1.48	<0.001	
Teacher (14)	Combined	0.48	1.01	<0.001	0.53
	Female	0.32	0.71	0.005	
	Male	0.68	1.17	<0.001	
Self (14)	Combined	0.76	1.09	<0.001	0.33
	Female	0.75	1.02	0.022	
	Male	0.77	1.13	<0.001	
Co-Twin (14)	Combined	0.76	1.23	<0.001	0.47
	Female	0.68	0.97	0.025	
	Male	0.86	1.38	<0.001	

[a]Mean differences evaluated using adjusted Wald test

[b]Cohen's *d*

ASPD: antisocial personality disorder

Online Resource 3 (cont).

Table: Pearson correlations between different informants' impulsivity and total aggression scores

**Informant, age, score**	P12 Agg	T12 Imp	T12 Agg	T14 Imp	T14 Agg	S14 Imp	S14 Agg	Tw14 Imp	Tw14 Agg
P12 Imp	0.54	0.42	0.31	0.34	0.25	0.28	0.10	0.39	0.20
P12 Agg		0.21	0.26	0.15	0.15	0.16	0.13	0.22	0.27
T12 Imp			0.73	0.49	0.39	0.33	0.19	0.41	0.19
T12 Agg				0.36	0.36	0.26	0.18	0.33	0.22
T14 Imp					0.68	0.39	0.20	0.47	0.24
T14 Agg						0.27	0.18	0.33	0.21
S14 Imp							0.38	0.51	0.21
S14 Agg								0.23	0.33
Tw14 Imp									0.47

P: parent rating; T: teacher rating; S: self rating; Tw: co-twin rating; Imp: impulsivity; Agg: aggression

Online Resource 3 (cont).

Table: Logistic regression models and AUC values for ASPD prediction by each informant's standardized impulsivity score, adjusted for sex and age

Informant (age of participant)	Model Variable	OR	95% CIs	AUC
Parent (12)				0.65
	Impulsivity	1.4*	1.1, 1.8	
	Sex	2.1*	1.2, 3.6	
Teacher (12)				0.67
	Impulsivity	1.6*	1.3, 2.0	
	Sex	1.5	0.9, 2.5	
Teacher (14)				0.73
	Impulsivity	1.7*	1.4, 2.1	
	Sex	1.6	0.8, 2.9	
Self (14)				0.72
	Impulsivity	1.8*	1.5, 2.3	
	Sex	1.9*	1.1, 3.1	
Co-Twin (14)				0.71
	Impulsivity	2.0*	1.6, 2.5	
	Sex	1.5	0.9, 2.5	

ASPD: antisocial personality disorder; CI: confidence interval; OR: odds ratio; AUC: area under the (receiver operating characteristic) curve

Online Resource 3 (cont).
Table: Logisitic regression models for ASPD prediction by each informant's standardized aggression (with impulsivity effect removed) or impulsivity (with aggression effect removed) score, adjusted by sex and age

Informant (age of participant)	Aggression (residual) scores				Impulsivity (residual) scores			
	Model Variable	OR	95% CIs	AUC	Model Variable	OR	95% CIs	AUC
Parent (12)				0.64				0.64
	Residual variable	1.1	0.9, 1.4		Residual variable	1.3	1.0, 1.6	
	Sex	2.3*	1.4, 4.0		Sex	2.2*	1.3, 3.8	
Teacher (12)				0.63				0.61
	Residual variable	1.2	1.0, 1.6		Residual variable	1.2	0.9, 1.6	
	Sex	2.3*	1.3, 3.8		Sex	1.9*	1.1, 3.2	
Teacher (14)				0.65				0.64
	Residual variable	1.4*	1.1, 1.8		Residual variable	1.1	0.9, 1.5	
	Sex	2.2*	1.2, 4.0		Sex	2.2*	1.2, 4.0	
Self (14)				0.68				0.67
	Residual variable	1.6*	1.3, 2.0		Residual variable	1.5*	1.2, 1.9	
	Sex	1.5	0.9, 2.6		Sex	2.0*	1.2, 3.4	
Co-Twin (14)				0.63				0.67
	Residual variable	1.3*	1.0, 1.7		Residual variable	1.6*	1.3, 2.1	
	Sex	1.8*	1.0, 3.2		Sex	1.8*	1.0, 3.1	

*$p<0.05$
Abbreviations: ASPD=antisocial personality disorder; CI=confidence interval; OR=odds ratio; AUC=area under the (receiver operating characteristic) curve

Online Resource 3 (cont).

Table: Multiple informant logistic regression model combination of impulsivity and total aggression scores for ASPD prediction, adjusted by sex and age

Model[a]	Model Variable	OR	95% CIs	AUC
All aggression + P12, S14, Tw14 impulsivity ratings	P12A	1.2	0.9, 1.6	0.82
	T12A	1.2	0.9, 1.7	
	T14A	1.5*	1.1, 2.0	
	S14A	1.5*	1.1, 2.1	
	Tw14A	1.0	0.7, 1.4	
	P12I	1.0	0.7,	
	S14I	1.2		
	Tw14I	1.3		
	Sex	1.2	0.6, 2.7	

P: parent rating; T: teacher rating; S: self rating; Tw: co-twin rating; A: aggression; I: impulsivity; ASPD: antisocial personality disorder; CI: confidence interval; OR: odds ratio; AUC: area under the (receiver operating characteristic) curve

[a]This model is significantly different (i.e., 'better'; p=0.015) than the PR12 + TR12 + TR14 + SR14 + TWR14 model in the main text Table 4.

Online Resource 4. Alternative anxiety rating logistic regression model for ASPD prediction

**Models with anxiety ratings (instead of aggression), adjusted for sex and age**						
**Model**	**N**	**Model Variable**	**OR**	**95% CIs**	**AUC**	**95% CIs**
Parent (12)	1278				0.61	0.54, 0.68
		Anxiety Rating	0.9	0.7, 1.2		
		Sex	2.3*	1.4, 3.9		
Teacher (12)	1301				0.63	0.56, 0.70
		Anxiety Rating	0.8	0.6, 1.0		
		Sex	2.2*	1.3, 3.7		
Teacher (14)	1016				0.61	0.53, 0.70
		Anxiety Rating	0.9	0.7, 1.2		
		Sex	2.2*	1.2, 4.0		
Self (14)	1312				0.60	0.54, 0.66
		Anxiety Rating	0.9	0.7, 1.2		
		Sex	1.8*	1.1, 3.1		
Co-twin (14)	1299				0.60	0.54, 0.67
		Anxiety Rating	0.9	0.7, 1.1		
		Sex	2.0*	1.2, 3.4		

*p<0.05
Abbreviations: ASPD=antisocial personality disorder; CI=confidence interval; OR=odds ratio; AUC=area under the (receiver operating characteristic) curve

Online Resource 5. Alternative ASPD diagnostic cut-off logistic regression models

**ASP diagnosis if 1+ criteria**						
**Model**	**N**	**Model Variable**	**OR**	**95% CIs**	**AUC**	**95% CIs**
Parent rating (12)	1278	Total Aggression Rating	1.3*	1.2, 1.5	0.63	0.57, 0.70
		Sex	1.8*	1.4, 2.3		
Teacher rating (12)	1302	Total Aggression Rating	1.4*	1.3, 1.6	0.68	0.60, 0.75
		Sex	1.7*	1.3, 2.1		
Teacher rating (14)	1013	Total Aggression Rating	1.3*	1.1, 1.5	0.70	0.62, 0.79
		Sex	1.7*	1.3, 2.3		
Self-rating (14)	1312	Total Aggression Rating	1.3*	1.2, 1.5	0.69	0.62, 0.76
		Sex	1.6*	1.3, 2.1		
Co-twin rating (14)	1208	Total Aggression Rating	1.5*	1.3, 1.7	0.68	0.60, 0.75
		Sex	1.6*	1.2, 2.0		
**ASP diagnosis if 2+ criteria**						
**Model**	**N**	**Model Variable**	**OR**	**95% CIs**	**AUC**	**95% CIs**
Parent rating (12)	1278	Total Aggression Rating	1.3*	1.1, 1.5	0.64	0.57, 0.71
		Sex	1.7*	1.2, 2.5		
Teacher rating (12)	1302	Total Aggression Rating	1.4*	1.2, 1.6	0.68	0.61, 0.75
		Sex	1.6*	1.1, 2.2		
Teacher rating (14)	1013	Total Aggression Rating	1.4*	1.2, 1.7	0.71	0.63, 0.80
		Sex	1.7*	1.1, 2.6		
Self-rating (14)	1312	Total Aggression Rating	1.6*	1.3, 1.8	0.71	0.64, 0.78
		Sex	1.4*	1.0, 2.0		
Co-twin rating (14)	1208	Total Aggression Rating	1.6*	1.3, 1.8	0.68	0.60, 0.76
		Sex	1.6*	1.1, 2.3		

Online Resource 5 (cont.)

**ASP diagnosis if 4+ criteria**						
**Model**	**N**	**Model Variable**	**OR**	**95% CIs**	**AUC**	**95% CIs**
Parent rating (12)	1278	Total Aggression Rating	1.4*	1.1, 1.8	0.65	0.59, 0.71
		Sex	2.5*	1.2, 5.2		
Teacher rating (12)	1302	Total Aggression Rating	1.5*	1.2, 1.8	0.68	0.62, 0.75
		Sex	2.2*	1.1, 4.4		
Teacher rating (14)	1013	Total Aggression Rating	1.6*	1.3, 2.0	0.72	0.64, 0.79
		Sex	2.2	0.9, 5.2		
Self-rating (14)	1312	Total Aggression Rating	2.0*	1.6, 2.5	0.72	0.66, 0.79
		Sex	1.4	0.7, 2.9		
Co-twin rating (14)	1208	Total Aggression Rating	1.8*	1.3, 2.4	0.69	0.62, 0.76
		Sex	1.7	0.8, 3.7		

**Subclinical cases (1-2 ASPD criteria) removed**						
**Model**	**N**	**Model Variable**	**OR**	**95% CIs**	**AUC**	**95% CIs**
Parent rating (12)	818	Total Aggression Rating	1.4*	1.2, 1.7	0.69	0.63, 0.76
		Sex	2.8*	1.6, 4.8		
Teacher rating (12)	842	Total Aggression Rating	1.8*	1.4, 2.2	0.72	0.66, 0.79
		Sex	2.1*	1.2, 3.6		
Teacher rating (14)	664	Total Aggression Rating	1.8*	1.4, 2.2	0.75	0.68, 0.83
		Sex	2.1*	1.1, 3.9		
Self-rating (14)	848	Total Aggression Rating	2.1*	1.6, 2.6	0.75	0.68, 0.82
		Sex	1.7	1.0, 2.9		
Co-twin rating (14)	773	Total Aggression Rating	1.9*	1.5, 2.4	0.73	0.66, 0.80
		Sex	2.0*	1.1, 3.6		

*p<0.05

Abbreviations: ASPD=antisocial personality disorder; CI=confidence interval; OR=odds ratio; AUC=area under the (receiver operating characteristic) curve

Online Resource 6. Logisitic regression models for ASPD prediction by each informant's standardized direct (with indirect aggression effect removed) or indirect (with direct aggression effect removed) aggression score, adjusted by sex and age

Informant (age of participant)	N	Direct (residual) aggression score					Indirect (residual) aggression score				
		Model Variable	OR	95% CIs	AUC	95% CIs	Model Variable	OR	95% CIs	AUC	95% CIs
Parent (12)	1277				0.66	0.60, 0.72				0.61	0.55, 0.67
		Residual variable	1.4*	1.1, 1.8			Residual variable	0.9	0.7, 1.1		
		Sex	2.0*	1.2, 3.5			Sex	2.3*	1.3, 3.9		
Teacher (12)	1292				0.67	0.60, 0.74				0.61	0.54, 0.67
		Residual variable	1.7*	1.3, 2.1			Residual variable	0.9	0.6, 1.3		
		Sex	1.4	0.8, 2.4			Sex	2.0*	1.1, 3.5		
Teacher (14)	994				0.69	0.60, 0.77				0.60	0.51, 0.69
		Residual variable	1.6*	1.2, 2.1			Residual variable	1.1	0.8, 1.6		
		Sex	1.6	0.8, 3.2			Sex	2.4*	1.3, 4.5		
Self (14)	1307				0.64	0.57, 0.72				0.65	0.58, 0.72
		Residual variable	1.4*	1.2, 1.8			Residual variable	1.4*	1.1, 1.7		
		Sex	1.6	0.9, 2.7			Sex	1.9*	1.1, 3.2		
Co-Twin (14)	1201				0.66	0.58, 0.74				0.60	0.54, 0.66
		Residual variable	1.6*	1.3, 2.0			Residual variable	1.1	0.6, 1.7		
		Sex	1.5	0.9, 2.6			Sex	2.0*	1.2, 3.5		

*p<0.05

Abbreviations: ASPD=antisocial personality disorder; CI=confidence interval; OR=odds ratio; AUC=area under the (receiver operating characteristic) curve

Online Resource 7. Attrition analysis comparing total aggression rating means, standard deviations (SD), and ranges for different sample sizes and data collection timepoints

Sample	Rating	N	Mean	SD	Range
All available FinnTwin12 participants (combined)	Parent (12)	4889	0.59	0.41	0-3
	Teacher (12)	4589	0.62	0.64	0-3
All FinnTwin12 partipants (except the 1347 in the final sample)	Parent (12)	3610	0.59	0.42	0-3
	Teacher (12)	3284	0.62	0.64	0-3
All available age 14 Intensive participants	Parent (12)	1745	0.60	0.41	0-2.5
	Teacher (12)	1782	0.65	0.65	0-3
All available age 22 Intensive participants	Parent (12)	1279	0.59	0.40	0-2.3
	Teacher (12)	1305	0.63	0.63	0-3
Those age 22 intensives w/only one twin available for age 22 even though both twins participated in age 14 intensive evaluation	Parent (12)	90	0.60	0.46	0-2.3
	Teacher (12)	94	0.68	0.73	0-2.8
Those age 22 intensives w/both twins available for both age 14 and age 22 intensive evaluations	Parent (12)	1168	0.58	0.39	0-2.3
	Teacher (12)	1190	0.63	0.63	0-3
